# Psychosocial developmental trajectory of a cohort of young adults born very preterm and/or with a very low birth weight in the Netherlands

**DOI:** 10.1186/s41687-019-0106-5

**Published:** 2019-03-07

**Authors:** Sylvia M. van der Pal, Heleen Maurice-Stam, Martha A. Grootenhuis, Aleid G. van Wassenaer-Leemhuis, Gijsbert H. W. Verrips

**Affiliations:** 1 TNO, Child Health, PO Box 3005, 2301 Leiden, DA the Netherlands; 2 Psychosocial Department, Emma Children’s Hospital, University Medical Center, Amsterdam, Netherlands; 3 Princess Máxima Center for paediatric oncology, Utrecht, Netherlands; 4 Department of Neonatology, Emma Children’s Hospital, Amsterdam University Medical Center, Amsterdam, Netherlands

**Keywords:** Preterm born, Young adults, Psychosocial, Developmental milestones, Course of life

## Abstract

**Background:**

The achievement of age-specific developmental milestones in youth is of great importance to the adjustment in adult life. Young adults who were born preterm, might go through a different developmental trajectory and transition into adulthood than their peers. This study aimed to compare the psychosocial developmental trajectory of young adults who were born preterm with peers from the general population. Young adults from the POPS (Project On Preterm and Small for gestational age infants) cohort study, born in 1983 in the Netherlands, completed online the Course of Life Questionnaire (CoLQ - achievement of psychosocial developmental milestones) at 28 years of age. Analysis of variance by group, age and gender was performed to test differences on the CoLQ scale scores between the POPS-group and 211 peers (25–30 years) from the general population (Ref-group). Differences on item level, representing the achievement of individual milestones, were analyzed with logistic regression analyses by group, age and gender.

**Results:**

The POPS-group (*n* = 300, 32,3% biased response) scored significantly lower than the Ref-group on the scales Psychosexual Development (effect size − 0.26, *p* < 0.01), Antisocial Behavior (ES − 0.44, *p* < 0.001) and Substance Use & Gambling (ES − 0.35, *p* < .001). A further exploration on item-level revealed, among others, that the POPS-group had their first boyfriend/girlfriend at later age, were more often single, misbehaved less at school and smoked, drank and gambled less than the Ref-group. On the scales Autonomy Development and Social Development no differences were found between the POPS-group and the Ref-group.

**Conclusions:**

A relatively less vulnerable respondent group of young adults born preterm showed some psychosocial developmental trajectory delays and might benefit from support at teenage age. Because of the non-response bias, we hypothesize that the total group of young adults born preterm will show more severe psychosocial developmental problems.

## Background

Improved perinatal care over the last decades has resulted in increased survival of infants who are born very preterm or with a very low birth weight [31]. As a result, an increased proportion of young adults who were born preterm may suffer from comorbidity because of their preterm birth.

The results of the Dutch nationwide POPS (Project On Preterm and Small for gestational age infants) cohort study of infants born very preterm and/or with very low birth weight in 1983, show a variety of follow-up outcomes until the age of 19 years. The POPS-19 study showed impairments on functional outcomes [15] and physical outcomes, such as blood pressure and insulin resistance [7, 18, 19] but also on risk-taking behavior and involvement in romantic relationships [14]. Other international studies show that on average young adults with very low birth weight are delayed in leaving the parental home and starting sexual activity and romantic relationships [17], have abnormal personality styles [1] and experience more psychiatric morbidity and depression, especially those born Small for Gestational Age (SGA) after intrauterine growth retardation [23, 27].

These findings indicate that young adults who were born preterm, might go through a different developmental trajectory and transition into adulthood than their peers, as also seen in young adults with a history of pediatric disease [30]. The fulfilling of age-specific developmental tasks in youth, as expressed in the achievement of psychosocial developmental milestones, is of great importance to the adjustment in adult life. With regard to autonomy development, obtaining a paid job is a one of the steps towards independence which most healthy youth put during secondary school. It is also common in that period of life that they search for their social contacts outside the family by spending most of their leisure time with friends (social development). And regarding psychosexual development, most young people have already been in love when they were eighteen [29]. A delayed psychosocial developmental trajectory (‘course of life’) may affect quality of life and socio-demographic outcomes in adulthood [8, 20, 24]. From a developmental psychological point of view, risk behavior is also relevant. To some extent, displaying risk-taking behavior - in terms of “experimenting with” - is part of the development of teenagers.

The current study aimed to assess the achievement of psychosocial developmental milestones while growing up in young adults being born very preterm and/or with very low birth weight, compared with young adults from the general population.

## Methods

### Subjects

#### POPS-group, young adults born preterm in 1983 of the nationwide POPS cohort

The POPS (Project On Preterm and Small for gestational age infants) cohort included 1.338 live-born, very preterm (gestational age < 32 weeks) and/or with very low birth weight (< 1500 g) infants born in the Netherlands in the year 1983 [38]. Previous data of the POPS cohort was collected at birth and ages two, five, nine, 10, 14 and 19 years [33]. In total, 381 (28.5%) of the POPS cohort participants did not survive to their 28th birthday, and 29 of them were lost to follow-up due to moving abroad or by specifically indicating no further interest to participate in the POPS studies in the future. As a result, 928 adults were eligible to participate in the current follow-up study at 28 years of age [36]. In the year they would turn 28, the year of 2011, participants of the POPS cohort were invited to participate in the POPS-28 online questionnaire follow-up study, either through an email or a letter [36]. In total 300 participants (32.3% of the POPS cohort who were eligible at age 28) completed the questionnaire. As presented in Table 1, non-participants were more often male and non-Caucasian, and they had a lower parental educational level and Social Economic Status (SES) than participants. In addition, at age 14 years, severe disabilities were more frequent in non-participants than in participants.

**Table 1:** Demographic and Neonatal characteristics: participants versus non-participants

	Total		Participants		Non-participants		Participants vs non-participants
	%	N	*%*	N	*%*	N	*p*-value
Gender							0.000
Female	*48.2*	461	*62.7*	188	*41.6*	273,384	
Male	*51.8*	496	*37.3*	112	*58.4*		
*Origin*							0.000
Caucasian	*85.3*	810	*93.6*	278	*81.5*	532	
Non-Caucasian	*14.7*	140	*6.4*	19	*18.5*	121	
Highest educational level parents ^a^,^b^							
Low	*38.9*	340	*26.8*	78	*44.9*	262	0.000
Middle	*36.3*	317	*39.2*	114	*34.8*	203	
High	*24.8*	217	*34.0*	99	*20.2*	118	
Social economic level parent ^c^							0.000
Very low	*10.1*	95	*3.4*	10	*13.3*	85	
Low	*32.3*	302	*27.2*	81	*34.6*	221	
Bit low	*22.0*	206	*22.1*	66	*21.9*	140	
Bit high	*9.0*	84	*10.4*	31	*8.3*	53	
High	*16.2*	152	*21.5*	64	*13.8*	88	
Very high	*10.4*	97	*15.4*	46	*8.0*	51	
Maternal age at time of birth (yrs) ^d^							
Mean (SD)	27.2 (4.9)		28.2 (4.3)		26.8 (5.0)		0.000
range	13.3–43.6		18.0–42.6		13.3–43.6		
< 20	*5.2*	49	*0.7*	2	*7.2*	47	0.000
≥ 20 and < 36	*90.9*	855	*94.5*	275	*89.2*	580	
≥36	*3.9*	37	*4.8*	14	*3.5*	23	
*Birth weight (grams)*							
Mean (SD)	1313.3 (282.7)		1312.5 (305.9)		1313.7 (271.7)		0.951
range	560–2580		560–2580		600–2400		
≤ 1000	*14.5*	139	*14.7*	44	*14.5*	95	0.353
1001–1250	*27.0*	258	*28.3*	85	*26.3*	173	
1251–1500	*38.6*	369	*34.7*	104	*40.3*	265	
> 1500	*20.0*	191	*22.3*	67	*18.9*	124	
Gestational age (weeks) ^e^							
Mean (SD)	31.0 (2.5)		31.0 (2.3)		31.1 (2.6)		0.534
range	25.4–40.9		26.0–37.6		25.4–40.9		
*Appropriate for gestational age (*yes*)* ^e^	*62.8*	600	*61.2*	183	*63.6*	417	0.516
Mechanical ventilation (days) ^f^							
Mean (SD)	4.8 (9.5)		4.7 (8.0)		4.8 (10.1)		0.823
range	0.0–117.0		0.0–43.0		0.0–117.0		
None	*0.0*	0	*0.0*	0	*0.0*	0	0.741
1–7	*79.1*	756	*78.3*	235	*79.4*	521	
8–28	*17.5*	167	*18.7*	56	*16.9*	111	
> 28	*3.5*	33	*3.0*	9	*3.7*	24	
*IRDS (yes)*	*39.0*	373	*42.3*	127	*37.4*	246	0.154
*ICH (yes)*	*17.2*	165	*19.0*	57	*16.4*	108	0.356
Disability at age 14 ^g^							0.029
none	*49.8*	421	*51.9*	149	*48.7*	272	
impairment (1 or 2 small)	*27.7*	234	*30.3*	87	*26.3*	147	
mild disability (> 2 small or 1 large)	*16.3*	138	*14.6*	42	*17.2*	96	
severe disability (> 1 large)	*6.3*	53	*3.1*	9	*7.9*	44	

Participants: Survivors of the POPS cohort at age 28 who completed the Course of Life Questionnaire

Non-participants: Survivors of the POPS cohort at age 28 who did not complete the Course of Life Questionnaire

^a^Low = primary education, lower vocational education; Middle = general secondary education, middle vocational education; High = higher vocational education, university

IRDS: idiopathic respiratory distress syndrome; ICH: intracerebral hemorrhage

^b^83 unknown: participants 9, non-participants 74

^c^21 unknown: participants 2, non-participants 19

^d^16 unknown: participants 9, non-participants 7

^e^2 unknown: participants 2, non-participants 0

^f^1 unknown: participants 0, non-participants 1

^g^111 unknown: participants 13, non-participants 98

Most participants completed the questionnaires online (*n* = 289), a small group completed the questionnaire on paper on request (*n* = 11). The medical ethics committee of the Leiden University Medical Center approved the POPS-28 study protocol. All POPS-28 participants sent in their written informed consent to participate in the study prior to the assessment.

#### Reference group (ref-group)

The reference group consists of young adults from the general Dutch population, who were participating as controls in a former study of the Emma Children’s hospital AMC on survivors of childhood cancer and were recruited through general practitioners [28]. The inclusion criteria were: (1) age at study 18–30 years, (2) no history of cancer, and (3) ability to understand questionnaires in Dutch. Results from previous research indicated that the Ref-group was representative for the general population of Dutch young adults, except for educational level and country of birth. Highly educated young adults and those born in the Netherlands were slightly over-represented [9], as is the case in most studies using written questionnaires. a.

For the current study, the young adults who were aged 25 to 30 were selected as reference group (Ref-group; *n* = 211). It was necessary to include reference respondents in a broader age-range than the age of 28 of the POPS-group to obtain a large enough sample size. A broader age-range was a sound choice as young adults between 25 and 30 years can be considered to be in the same phase of life. The medical ethical committee of the Academic Medical Center (AMC) approved the study protocol.

### Measures

#### Course of life questionnaire to measure psychosocial developmental milestones

The Course of Life Questionnaire (CoLQ) was used to assess the achievement of psychosocial developmental milestones while growing up [9, 28]. This instrument was developed by the Psychosocial Department of the Emma Children’s hospital AMC to be able to investigate the psychosocial developmental trajectory (CoL) of young adults who have grown up with a chronic or life-threatening disease, and to facilitate comparison with peers from the general population. The items, based on the literature and on clinical experience, concern behavior characteristic of certain age stages, psychosocial developmental tasks, and the limitations children might face when they grow up with a chronic disease. Most questions ask retrospectively whether the respondent had achieved certain psychosocial milestones or at what age the respondent achieved the milestones. The answers are dichotomized (1 = milestone not achieved, 2 = milestone achieved), if necessary, before being added up to the scale-score. The items are divided into five scales: autonomy development (6 items, autonomy at home and outside the home), psychosexual development (4 items, love and sexual relations), social development (12 items, contacts with peers), antisocial behavior (4 items, misbehavior at school and outside it), and substance use and gambling (12 items, gambling, use of tobacco, alcohol, soft drugs, psychedelic drugs and hard drugs). A higher score on the first three scales indicates the accomplishment of more psychosocial developmental milestones. Higher scores on the scales of antisocial behavior and on substance use and gambling mean that the respondent displays more risk behavior.

The psychometric characteristics of the CoL-scales are satisfactory, including reliability and validity. The internal consistency of the scales was moderate to good as expressed by the following Cronbach’s alphas in the reference group of young adults (18–30 years): autonomy 0.49, social development 0.79, psychosexual development 0.71, antisocial behavior 0.57, substance use and gambling 0.78 [9, 28]. The CoLQ has been used in the general Dutch population of young adults and in young adults with diverse chronic diseases since childhood [2–5, 10, 11, 13, 16, 21, 22, 25, 28, 30, 34, 35, 37]. Data from the general Dutch population served as reference data for the current study (see SUBJECTS, Reference group).

Apart from the five scales, socio-demographic variables were measured: marital status (living with a partner versus single), educational level of participants and their parents (low, middle or high; based on the highest level of education completed), and employment status (employed versus unemployed).

### Statistical methods

Differences in the characteristics of participants versus non-participants and POPS- versus the Ref-group were tested with the Chi2-test (gender, educational level, employment status) and t-test (age). To assess the internal consistency of the scales of the CoLQ, Cronbach’s alphas were calculated for the POPS-group and the Ref-group.

Analysis of variance (ANOVA) by group, age and gender was performed to test differences on the scale scores of the CoLQ between POPS- and Ref-group. The educational level and marital status of the participants were not included as a covariates because these can be considered to be outcomes. Handicaps at 5 years were not included because data was not available for the Ref-group. It was not necessary to correct the analyses for country of birth as ‘born in the Netherlands’ (yes/no) was not associated with the outcomes. Because of multiple scales, and therefore multiple testing, a significance level of 0.01 was used; 0.05 divided by the number of five scales. Effect sizes were calculated by dividing the difference in mean scores between POPS- and the Ref-group by the pooled standard deviation. Effect sizes up to 0.2 were considered small, effect sizes of 0.2–0.5 are considered small to medium, effect sizes of 0.5–0.8 are considered medium to large and effect sizes > 0.8 are considered large [6]. In addition, non-parametric Mann-Whitney U tests were performed, since the scale scores were not distributed quite normally, and confirmed the results found with the ANOVA (Analysis of Variance). Therefore, in this article the results of the ANOVA are reported.

In order to gain more detailed insight into the developmental trajectory, additional differences on item level – indicating the achievement of a milestone – were analyzed with logistic regression analyses by group, age and gender, including Odds ratios (ORs) for POPS- vs Ref-group. A significance level of 0.05 was used. Only the items for which significant differences were found between POPS and Ref-group, are displayed in Table 3. The Statistical Package for the Social Sciences (SPSS) version 20 was used for all analyses.

## Results

### Socio-demographic characteristics

Socio-demographic characteristics of the POPS-group and the Ref-group were presented in Table 2. The educational level of the young adults from the POPS-group as well as the educational level of their parents were higher than in the Ref-group (*p* < 0.01, *p* < 0.001 respectively). In the POPS-group significantly more young adults were single than the Ref-group (*p* < 0.01).

**Table 2:** Socio-demographic characteristics

	POPS (n = 300)	Ref (n = 211)
Age (yrs)		
Mean	27.97	28.14
SD	0.28	1.75
Range	27.47–28.59	25.04–30.96
	*% (N)*	*% (N)*
*Gender*: female	63.0 (189)	55.0 (116)
*Country of birth*: the Netherlands	100 (300)	94.8 (199)
Educational level parents ^a,d^		
Low	33.9 (96)	53.5 (108)
Middle	24.4 (69)	25.2 (51)
High	41.7 (118)	21.3 (43)
Marital status participant ^b^		
Married / Living together	52.0 (156)	66.5 (139)
Single	48.0 (144)	33.5 (70)
Educational level participant ^c^,^d^		
Low	16.1 (48)	28.1 (57)
Middle	37.9 (113)	35.0 (71)
High	46.0 (137)	36.9 (75)
Paid job		
Yes	87.0 (261)	90.9 (190)
No	13.0 (39)	9.1 (19)

^a^POPS vs Ref: *p* < 0.001 (according to Chi-square test)

^b^POPS vs Ref: *p* < 0.01 (according to Logistic regression analysis by group, age and gender)

^c^POPS vs Ref: p < 0.01 (according to Logistic regression analysis by group, age and gender)

^d^Highest educational level completed: Low: Primary Education, Technical and Vocational Training, Lower and Middle General Secondary Education. Middle: Middle Vocational Education, Higher General Secondary Education, Pre-university Education. High: Higher Vocational Education, University

### Internal consistency of the scales of the ColQ

In the current study, the internal consistency of the scales was moderate to good as expressed by the following Cronbach’s alphas: autonomy POPS-group 0.52, Ref-group 0.56; social development POPS-group 077, Ref-group 0.69; psychosexual development POPS-group 0.75, Ref-group 0.76; antisocial behavior POPS-group 0.51, Ref-group 0.49; substance use and gambling POPS-group 0.79, Ref-group 0.77.

### Autonomy development

The mean scale score on Autonomy Development of the POPS-group did not differ significantly from the mean scale score of the Ref-group (Table 3). Additional analyses on item level (Table 4) showed also no significant difference between the POPS-group and the Ref-group.

**Table 3:** Psychosocial development trajectory of POPS-group vs Ref-group: scale scores Course of life questionnaire

	POPS		Ref		POPS vs Ref
	*N*	*M(SD)*	*N*	*M(SD)*	*Effect size d*
Autonomy development	300	9.7 (1.4)	207	9.6 (1.5)	0.09
Social development	297	20.5 (2.9)	198	20.7 (2.5)	- 0.08
Psychosexual development	299	6.8 (1.3)	209	7.1 (1.2)	- 0.26*
Antisocial behavior	300	4.3 (0.7)	210	4.7 (0.9)	- 0.44**
Substance use and gambling	300	14.1 (2.4)	204	15.0 (2.5)	- 0.35**

*Differences at p < 0.01 according to ANOVA by group, age and gender

**Differences at *p* < 0.001 according to ANOVA by group, age and gender

**Table 4:** Psychosocial development trajectory of POPS-group vs Ref-group: significant differences on item-level (milestones) Course of life questionnaire

	POPS		Ref		POPS versus Ref
	*%*	*N*	*%*	*N*	*OR (95%-CI)*
Social development					
Most of the time playing with….., primary school					
- *Friends*	*77.3*	232	*85.1*	177	0.56*
- *Brothers and/or sisters, parents, on your own*	*22.7*	68	*14.9*	31	(0.34–0.91)
Psychosexual development					
First girlfriend / boyfriend					
- *At the age of 17 or younger*	*65.3*	196	*76.7*	161	0.55**
- *At the age of 18 or older/never*	*34.7*	104	*23.3*	49	(0.37–0.83)
For the first time sexual intimacy					
- *At the age of 18 or younger*	*74.7*	224	*83.4*	176	0.58*
- *At the age of 19 or older/never*	*25.3*	76	*16.6*	35	(0.37–0.90)
For the first time sexual intercourse					
- *At the age of 18 or younger*	*47.8*	143	*60.5*	127	0.57**
- *At the age of 19 or older/never*	*52.2*	156	*39.5*	83	(0.40–0.82)
Antisocial behavior					
Ever been suspended because of misbehavior at school, primary school					
- Yes	*1.7*	5	*7.1*	15	0.25**
- *No*	*98.3*	295	*92.9*	196	(0.09–0.70)
Get into trouble with the police or law, secondary school					
- *Yes*	*8.7*	26	*18.1*	38	0.47**
- *No*	*91.3*	274	*81.9*	172	(0.27–0.81)
Ever been suspended because of misbehavior at school, secondary school					
- *Yes*	*5.7*	17	*13.8*	29	0.41**
- *No*	*94.3*	283	*86.2*	181	(0.22–0.79)
Ever been refused admission to lessons, secondary school					
- *Yes*	*16.7*	50	*29.5*	62	0.50**
- *No*	*83.3*	250	*70.5*	148	(0.32–0.76)
Substance use and gambling					
Alcohol, secondary school					
- *Often / very often*	*16.7*	50	*26.2*	55	0.59*
- *Never / occasionally*	*83.3*	250	*73.8*	155	(0.38–0.92)
Smoking, secondary school					
- *Yes*	*25.0*	75	*35.9*	75	0.59**
- *No*	*75.0*	225	*64.1*	134	(0.40–0.87)
Gambling, secondary school					
- *Occasionally / often / very often*	*9.0*	27	*22.0*	46	0.36***
- *Never*	*91.0*	273	*78.0*	163	(0.21–0.62)
Smoking, after secondary school					
- *Yes*	*26.0*	78	*51.0*	107	0.35***
- *No*	*74.0*	222	*49.0*	103	(0.24–0.50)

*p < 0.05 according to Logistic regression analysis by group, age and gender

**p < 0.01 according to Logistic regression analysis by group, age and gender

***p < 0.001 according to Logistic regression analysis by group, age and gender

Only the items which the POPS-group differed significantly from the Ref-group are displayed this table

### Social development

The mean scale score on Social Development of the POPS-group did not differ significantly from the mean scale score of the Ref-group (Table 3). On item-level (Table 4), one significant difference between the POPS-group and Ref-group was found, on the item “Most of the time playing with …, in primary school”, where 77.3% of the POPS-group indicated that they played with friends most of the time, compared to 85.1% in the Ref-group (OR = 0.56, *p* < .05).

### Psychosexual development

The POPS-group scored significantly lower (mean = 6.8) than the Ref-group (mean = 7.1, p < 0.01) on the scale Psychosexual Development (Table 3). The additional analyses on item-level (Table 4) showed that the POPS-group had their first girl/boy-friend, first sexual intimacy and first sexual intercourse at later age than the Ref-group. The Odds Ratios ranged from 0.55 (*p* < .01) to 0.58 (p < .05).

### Antisocial behavior

The POPS-group scored significantly lower (mean = 4.3) than the Ref-group (mean = 4.7, *p* < 0.001) on the scale Antisocial Behavior (Table 3). The additional analyses on item-level (Table 4) revealed that the POPS-group showed significantly less problem behavior (misbehavior at school and getting in trouble with the police) on all four items than the Ref-group.

### Substance use & gambling

The POPS-group scored significantly lower (mean = 14.1) than the Ref-group (mean = 15.0, *p* < 0.001) on the scale Substance Use & Gambling (Table 3). With regard to alcohol use (Table 4), the POPS-group used less frequently alcohol during secondary school (16.7% often/very often) than the Ref-group (26.2%, *p* < 0.05). After secondary school, the frequency of alcohol use in the POPS-group (40.0% often/very often) was comparable to the Ref-group (48.6%). With regard to gambling, the same pattern was found. The POPS-group gambled less often than the Ref-group at secondary school (9% vs 22%, p < 0.001), while after secondary school, the frequency of gambling in the POPS-group (34.7%) was comparable to the Ref-group (41.7%). Finally, in the POPS-group fewer respondents smoked compared to the Ref-group; at secondary school 25.0% versus 35.9% (*p* < 0.01), after secondary school 26.0% versus 51.0% (p < 0.001). No significant differences were found on the six items regarding use of soft, psychedelic and hard drugs (data not shown).

## Discussion

This article presents an overview of the psychosocial developmental trajectory of young adults born very preterm or with very low birth weight in the POPS cohort in the Netherlands at 28 years of age, compared to controls from the general population. In line with the results regarding social lifestyle and risk-taking behavior of the POPS cohort at 19 years of age [14], the largest differences in psychosocial development between the POPS- and Ref-group were found in the domains of antisocial behavior, gambling and substance use and psychosexual development. Compared to the Ref-group, the domain scores of the POPS-group showed some delay in psychosocial development, expressed by small to medium effect sizes (d) with regard to: Antisocial behavior (d = − 0.44), Substance use and gambling (d = − 0.35) and Psychosexual development (d = − 0.26).

In the domain of Anti-social behavior, the largest difference was found on the item “suspended from primary school because of misbehavior”, which the POPS-group experienced less often (odds ratio of 0.25) than the ref-group. The POPS-group is also less likely to start gambling and use alcohol at secondary school, or start with this risk taking behavior later in life than their peers. This supports earlier findings of the POPS study at 19 years [14]. Not displaying risk-taking behavior could be seen as a protective factor, but displaying risk-taking behavior is also part of the normal developmental experiences of teenagers.

The delay found in the Psychosexual development of the POPS-group, including marital status, is also in line with the results of the POPS cohort at 19 years of age [14] and with a study from Finland, which also showed that young adults with very low birth weight had a delay in leaving the parental home and starting sexual activity and romantic relationships [17].

A very preterm birth or very low birth weight status may promote a more protective parenting style. The complicated birth and the vulnerability of the child after birth can result in parental perception of heightened general vulnerability of their child, which may lead parents to behave more indulgent, controlling, protective or intrusive [32]. In a study by [26] [26], parenting of mothers was rated as more protective and authoritarian by young adults born preterm than by controls born at term. The mothers and fathers of the preterm born young adults rated themselves higher on supportive parenting [26]. The question arises whether a delayed developmental trajectory and transition into adulthood is caused by physical or cognitive problems or by a different or overprotective parental attachment resulting from preterm birth [12, 26]. Future research should further investigate the underlying mechanisms.

There are limitations to this study. The fact that severe disabilities were more frequent in non-participants than in participants limits the generalizability of the results. Because of this non-response bias, our sample represents the better end of the spectrum of young adults born very preterm or with very low birth weight. So, the current study might display an underrepresentation of the psychosocial developmental problems of young adults born preterm. However, even in this study sample, the results showed some delays among the POPS group in the achievement of developmental milestones, compared to peers from the general population. Future research should reveal whether certain subgroups of young adults born preterm are at greater risk of delay in the achievement of psychosocial developmental milestones. In a study among survivors of childhood cancer, it was found that having been treated for a brain tumor was related to delay in the developmental trajectory, indicating that cognitive problems might influence the achievement of psychosocial developmental milestones negatively [24, 25, 28].

The demographics in Table 1 and Table 2 show that in the POPS-group those with highly educated parents were overrepresented, which suggests a non-response bias, possibly reflecting the low response rate. Controlling the analyses for parental level of education would not have yield other results, as we found that the parental level of education was only weakly correlated with the young adult’s CoL scores (data not shown). The young adults in the POPS-group were more highly educated and more often single than the Ref-group. However, controlling the analyses for educational level and marital status of the young adults was not appropriate because the level of education and marital status could be considered as outcomes of being born very preterm. Nevertheless, we conducted additional analyses with inclusion of educational level and marital status of the young adults as covariates, which did not yield other results (data not shown) than the analyses without these covariates as reported in this paper. Finally, some limitations of the ColQ should be mentioned. First, the psychosocial developmental trajectory is more comprehensive than covered by the CoLQ. To prevent recall-bias, both in the POPS-group and the Ref-group, only ‘factual’ milestones are assessed with the CoLQ and the milestones do not go further back than to the period of primary school. Although this reduces the risk of recall-bias, results should be interpreted with caution because recall-bias could not be ruled out completely. Second, though we used Cohen’s effect sizes d to interpret differences on the CoLQ scales, the interpretation of the differences was hampered by the lack of information about clinically important differences. In future research, efforts should be put into establishing the clinically important difference for CoLQ outcomes. The question arises whether the results in the POPS cohort born in 1983 are still being relevant for children being born premature nowadays. A comparison of the early life outcomes of the POPS cohort with a more recent Dutch cohort of children born with the same inclusion criteria showed that the children who survived, all experienced morbidities that the survivors in 1983 also faced [31] This indicates that the results from the present study may be still relevant for children who are born nowadays.

## Conclusions

In conclusion, a relatively less vulnerable respondent group of young adults born preterm showed some psychosocial developmental trajectory delays and might benefit from support at teenage age. Because of the non-response bias, we hypothesize that the total group of young adults born preterm will show more severe psychosocial developmental problems. This knowledge is important to make health care providers aware of possible gaps in psychosocial development in this group. In the Netherlands, public child health professionals are involved in the health care for children and adolescents. When these professionals encounter children or adolescents who were born preterm, they should be alert for delays in psychosocial development and support these teenagers in relational and societal participation and in their transition into adulthood.

## Abbreviations

ANOVA: Analysis of variance
CoL: Course of life; psychosocial developmental trajectory
CoLQ: Course of life questionnaire (− achievement of psychosocial developmental milestones)
ORs: Odds ratios
POPS: Project on preterm and small for gestational age infants cohort study
SD: Standard deviation
SES: Social economic status
SGA: Small for gestational age
SPSS: Statistical package for the social sciences
